# Progress of new-onset diabetes after liver and kidney transplantation

**DOI:** 10.3389/fendo.2023.1091843

**Published:** 2023-02-08

**Authors:** Zhen Zhang, Jianyun Sun, Meng Guo, Xuemin Yuan

**Affiliations:** ^1^ Department of Urology, The People’s Hospital of Linyi, Linyi, Shandong, China; ^2^ Department of Gastroenterology, The People’s Hospital of Linyi, Linyi, Shandong, China; ^3^ National Key Laboratory of Medical Immunology &Institute of Immunology, Navy Medical University, Shanghai, China

**Keywords:** liver transplantation, kidney transplantation, post transplantation diabetes mellitus, insulin resistance, hyperglycemia

## Abstract

Organ transplantation is currently the most effective treatment for end-stage organ failure. Post transplantation diabetes mellitus (PTDM) is a severe complication after organ transplantation that seriously affects the short-term and long-term survival of recipients. However, PTDM is often overlooked or poorly managed in its early stage. This article provides an overview of the incidence, and pathogenesis of and risk factors for PTDM, aiming to gain a deeper understanding of PTDM and improve the quality of life of recipients.

## 1 Introduction

Liver transplantation is currently the primary procedure for chronic end-stage liver disease and acute liver failure at present (1). Kidney transplantation is the most ideal therapy for end-stage renal disease at home and abroad (2). Post transplantation diabetes mellitus (PTDM) is a common severe complication after liver and kidney transplantation. Its occurrence can have serious consequences, eventually leading to a decrease in the survival rate of the graft (3), and seriously affecting the prognosis of the recipient after organ transplantation. The incidence of PTDM after liver transplantation is 9.8-63.3% (4, 5), and can reach 2.5-24.0% (6) after kidney transplantation.

## 2 Incidence of new-onset diabetes after transplantation

### 2.1 New-onset diabetes after liver transplantation

Liver transplantation is currently the best procedure for end-stage liver disease (7, 8). With development of medical technology and improvements in liver transplantation techniques, the number of people receiving liver transplantation has increased significantly (9, 10). With the development of liver transplantation techniques, the optimization of immunosuppressant regimens, and the improvement of perioperative monitoring, the incidence of short-term postoperative complications, such as postoperative acute rejection, has been significantly reduced, so the short-term survival rate has gradually increased. Currently, long-term complications and postoperative quality of life after liver transplantation are attracting increasing attention. Post transplantation metabolic complications have adverse effects on the survival rate of recipients and the function of transplants (11, 12). PTDM is the most common metabolic complication after liver transplantation (13, 14), and has a considerable impact on postoperative rejection, cardiovascular disease, infection, neuropsychiatric problems and other complications, thereby affecting the function of the transplanted kidney and the long-term quality of life of the recipients (11, 15–23). Qi Ling et al. (24) studied the data of 10,204 patients without preoperative diabetes in the China Liver Transplant Registry (CLTR) from 2000 to 2013, and found that the survival rate of patients with PTDM was significantly lower than the overall survival rate. Lv C (25) found that the average survival rate of the PTDM and non-PTDM groups was 4.22 ± 0.26 years old (95%CI3.71-4.73) in a retrospective study of 428 liver transplant recipients, while the average survival rate of the non-PTDM group was 6.13 ± 0.20 years (95%CI5.36-6.04). In addition, Moon et al. (20) analyzed the data of 778 liver transplant recipients and found that the survival rates of recipients and grafts in the PTDM group were significantly lower than those in the non-PTDM group (**Table 1**).

**Table 1:** Incidence of PTDM after liver transplantation (%).

Study	Type	Duration	Total number of cases (cases)	PTDM cases (cases)	Incidence (%)
Lv C (25)	Retrospective study	2001.4-2008.12	428	87	20.3
J.Zhao (26)	Retrospective study	2001-2008.3	74	11	14.9
Honda M (27)	Retrospective study	1998.12-2011.10	161	22	13.7
Van LaeckeS (28)	Retrospective study	2004-2009	169	52	30.8
SaabS (29)	Retrospective study	1998.1-2002.12	253	45	17.8
BaidS (30)	Retrospective study	1991.1-1998.12	111	41	37
Tueche SG (31)	Retrospective study	1998.1-2000.12	143	45	31
Terto SV (32)	Retrospective study	2011-2014	29	146	19.8

### 2.2 New-onset diabetes after kidney transplantation

Compared to dialysis, kidney transplantation is the best procedure for patients with end-stage renal disease (33–35). However, PTDM is a long-term complication after kidney transplantation. Compared to recipients with normal plasma glucose levels after surgery, kidney transplant recipients with PTDM have an increased risk of adverse outcomes. PTDM severely affects the quality of life and long-term survival of kidney transplant recipients (36–39), and its occurrence is caused by multiple factors. Studies have shown that age, BMI, preoperative hyperglycemia, genetic factors, acute rejection and the use of glucocorticoids are independent risk factors for PTDM (40–44). Through PTDM-related studies, it was found that the survival rate of recipients without PTDM after surgery was 98%, while that of recipients with PTDM was only 83%. Dienemann T et al. (45) followed up recipients with PTDM for up to 12 years and found that the graft survival rate of non-PTDM recipients was approximately 70%, while that of PTDM recipients was only 48%, and the risk of PTDM recipients having graft loss was 3.72 times higher than that of non-PTDM recipients. PTDM is also an independent risk factor for reducing the recipient survival rate (46). A retrospective study with a large sample of data from kidney transplant recipients diagnosed with PTDM during a 2-year follow-up period found that the incidences of renal, ocular, neurological and peripheral vascular complications in PTDM recipients were 31.3%, 8.3%, 16.2%, and 4.1%,respectively, and circulatory microvascular complications were common, suggesting that complications in PTDM recipients occurred and deteriorated more rapidly than in recipients with common T2DM or pretransplant diabetes mellitus (47) (**Table 2**).

**Table 2:** Incidence of PTDM after kidney transplantation (%).

Study	Type	Duration	Total number of cases (cases)	PTDM cases (cases)	Incidence (%)
Fang DONG (48)	Retrospective study	2013.1-2018.6	549	110	20
GulsoyKirnap N (49)	Retrospective study	2010-2019	400	62	15.5
L. Xie (50)	Retrospective study	2007.1-2010.5	397	37	9.3
GomesV (51)	Retrospective study	2012.1-2016.3	125	34	27.2
SinangilA (52)	Retrospective study	2005.2-2014.2	420	70	16.6
Bzoma B (53)	Retrospective study	2001-2016	1424	109	7.6
AlagbeSC (54)	Retrospective study	2004-2008	111	20	18
Tomkins M (41)	Retrospective study	2006.1-2017.12	347	38	10.9

## 3 Pathogenesis and risk factors

Diabetes is caused by an absolute or relative deficiency of insulin secretion. The pathogenesis of PTDM is similar to that of type 2 diabetes. The main pathogenesis of PTDM is currently recognized as pancreatic β-cell dysfunction and insulin resistance, that is, decreased pancreatic β-cell secretion, compromised insulin sensitivity, and increased peripheral insulin resistance, resulting in impaired glucose tolerance; hence, the diagnostic criteria for diabetes are met. Studies (55, 56) have shown that impaired insulin secretion is the main pathogenesis of PTDM after kidney transplantation. However, Nagaraja et al. (57, 58) reported that insulin resistance is the main pathogenesis of PTDM. Studies have shown that insulin resistance and insulin deficiency work together to cause hyperglycemia after kidney transplantation (59). Fang Dong et al. (48) reported that the hypofunction of pancreatic β cells led to insufficient insulin secretion, making it difficult for patients with abnormal glucose metabolism to recover normal plasma glucose levels after kidney transplantation so that they eventually develop PTDM. Compared with type 2 diabetes, most PTDM can be reversed, and it is related to factors such as immunosuppressant regimen, age at transplantation, and recovery of islet β-cell function. Age is an independent predictor, older age at transplantation, is associated with a greater risk of irreversible postoperative PTDM.

The risk factors leading to PTDM are complex, and the predisposing factors and pathogenesis are often affected by multiple variables. The ability to predict a patient’s risk of PTDM is very beneficial for selecting an appropriate immunosuppressant regimen. Use of immunosuppressants, lifestyle changes, viral infections, recipient, donor, and surgery-related factors can all contribute to its occurrence. Transient hyperglycemia might be present in the early post transplantation and acute complications, and although transient hyperglycemia is excluded from the definition of PTDM, it is a risk factor for the occurrence of PTDM (60).

Huapeng Lu et al. (5) included 10,043 patients through a literature search. The results of the meta-analysis showed that: recipient age, sex, BMI, fasting plasma glucose (FPG) before transplantation, family history of diabetes, recipient HCV infection, recipient CMV infection, donor gender, liver steatosis, donor CMV infection, transplantation method, postoperative acute rejection, early infection after transplantation, and immunosuppressive agents were all risk factors for PTDM. In addition, some studies have divided the risk factors for PTDM into two types: modifiable and non-modifiable. Non-modifiable risk factors include race, age, sex, family book, HLA phenotype, deceased donor, autosomal dominant polycystic kidney disease (ADPKD) and impaired pre-transplantation FPG; modifiable risk factors include use of immunosuppressants, obesity, and post transplantation opportunistic infections (61).

## 4 Use of immunosuppressants

Immunosuppressive drugs, including glucocorticoids, calcineurin inhibitors, and mammalian target of rapamycin inhibitors, all play important roles in the development of PTDM (62–65), and are more vital than traditional risk factors.

## 5 The mechanism of glucocorticoid and tacrolimus in regulating pancreatic glucose metabolism (drawing with photoshop)

### 5.1 Glucocorticoids

**Figure 1:**
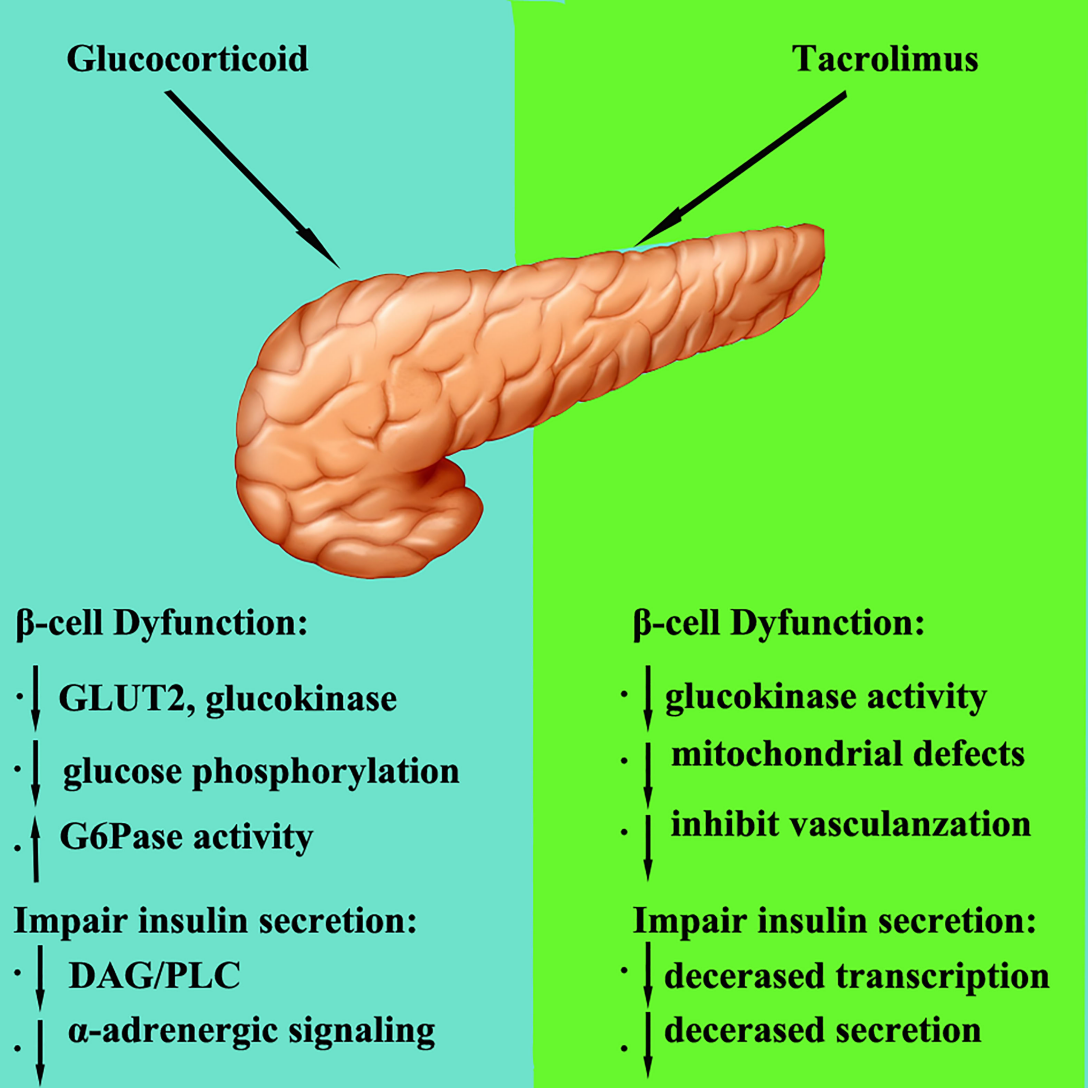
The mechanism of glucocorticoid and tacrolimus in regulating pancreatic glucose metabolism.

Glucocorticoids are widely used in the early stage after transplantation and when organ rejection occurs. The quantity of glucocorticoids used after transplantation is large, especially when acute rejection occurs, and glucocorticoid pulse therapy is needed. Glucocorticoids can interfere with glucose metabolism in different ways, thereby affecting plasma glucose levels (**Figure 1**). The main mechanisms by which glucocorticoids lead to abnormal glucose metabolism are as follows: Glucocorticoids not only inhibit the binding of insulin to its receptors, but also impair the post-receptor glucose transport system in peripheral tissues and inhibit the uptake and utilization of glucose in peripheral tissues; Glucocorticoids can promote gluconeogenesis and activate a variety of enzymes related to gluconeogenesis, thereby increasing the synthesis of liver glycogen and muscle glycogen; Toxic and anti-proliferative effects of glucocorticoids on islet β cells, affect the synthesis and secretion of insulin (66–69). Ranta et al. (70) found that large doses of glucocorticoids could induce pancreatic β-cell apoptosis by activating calcineurin and corticosteroid receptors, thereby reducing insulin secretion. It is widely believed that the main mechanism of corticosteroid induced PTDM is increased insulin resistance and weight gain (71). Studies (72) have found that glucocorticoid induced PTDM depends on the cumulative dose and duration of drug use.

### 5.2 CNIs

Calcineurin inhibitors (CNIs) include cyclosporine and tacrolimus, which inhibit calcineurin. Calcineurin is essential for the survival, replication, and function of β cells. Currently, it is believed that calcineurin inhibitors lead to an increase in plasma glucose mainly in the following ways (1): damage to pancreatic β cells; (2) and affecting of insulin secretion.

#### 5.2.1 Calcineurin inhibitors damage islet β cells

Calcineurin inhibitors inhibit calcineurin and its downstream pathways, reduce the survival and replication of β cells, and then affect insulin secretion (73). Studies reported that tacrolimus has a toxic, damaging effect on pancreatic β cells. Tacrolimus can degenerate pancreatic ducts, reduce the number of β cells, cause cytoplasmic swelling and vacuolization, and induce apoptosis, resulting in decreased insulin synthesis and secretion and insulin resistance (74, 75). Heit et al. (76) claimed that calcineurin inhibitors result in decreased β cells proliferation and a progressive decrease in β cells number. Tacrolimus not only impairs islet β cells function, but also induces insulin resistance (77).

#### 5.2.2 Calcineurin inhibitors reduce insulin secretion

Studies have reported that tacrolimus can reduce insulin secretion and insulin receptor expression by inducing pancreatic cell apoptosis, thereby increasing insulin resistance and plasma glucose concentrations to cause diabetes (78). Studies have confirmed that islet β cells contain a large amount of tacrolimus cytoplasmic receptor-FKBP-12, and high concentrations of FKBP-12 promote the aggregation of FK506 in β cells and their combination into the FK506-FKBP-12 complex, inhibiting calcineurin activity and the activation of glucose and the extracellular K^+^ insulin gene promoter, interrupting insulin mRNA transcription and reducing insulin secretion. Redmond et al. (79) and Radu et al. (80) revealed that CNIs at regular doses can disrupt insulin gene transcription, impair glucokinase activity, and reduce insulin reserves and ultimately insulin secretion. The progressive decrease in β cells and insulin secretion eventually leads to PTDM (81).

Cyclosporine can inhibit pancreatic islet cell function, reduce insulin secretion, and might also reduce the sensitivity of peripheral tissues to insulin (82). In addition, there have also been studies showing that the effect of tacrolimus is stronger than that of cyclosporine (64, 79), tacrolimus can decrease insulin secretion without altering insulin resistance, and its effect on insulin secretion is dose-dependent.

### 5.3 Mammalian target of rapamycin inhibitor

The mammalian target of the rapamycin inhibitor sirolimus has also been identified as a risk factor for PTDM, Johnston et al. (83) found that sirolimus was associated with an increased risk of PTDM with or without concurrent use of calcineurin inhibitors or antimetabolites. Teutonico et al. (84) found that insulin resistance increased, and insulin responsiveness decreased after calcineurin inhibitor withdrawal and conversion to sirolimus.

#### 5.3.1 Lifestyle changes

The lifestyle changes after liver transplantation mainly affect plasma glucose in the following two ways: on the one hand, the improvement of appetite and other factors lead to an increase in total calorie, carbohydrate and lipid intake, leading in turn to weight gain (> 20% or more than body weight before surgery), In addition, there is an increased incidence of central obesity, and weight gain is an important risk factor for diabetes and other metabolic syndromes, including dyslipidemia and hypertension (85). On the other hand, improved general health and increased protein intake following successful liver transplantation can reverse symptoms of muscle weakness and increase daily physical activity, which could have significant cardiometabolic benefits, in turn affecting plasma glucose levels.

Weight gain is common in recipients after liver transplantation, with 30-60% of them being overweight or obese, typically gaining an average of 2-9 kg within one year. One year after liver transplantation, weight gain usually slows (85–89). Mechanisms leading to post-transplantation weight gain are driven by a complex interplay of genetic, physiological, behavioral and environmental factors (90, 91). Dyslipidemia and hypertension occur in 40-70% of recipients, and are usually associated with the use of mammalian target of rapamycin (mTOR) inhibitors and calcineurin inhibitors.

The risk factors for PTDM are the same as those for type 2 diabetes, such as age > 45 years old, race, family history, history of gestational diabetes or macrosomia, poor lifestyle, and overweight or obesity. Studies have also reported that triglycerides and/or low high-density lipoprotein cholesterol, hypertension, and polycystic ovary syndrome (PCOS) also increase the risk of type 2 diabetes (92).

#### 5.3.2 Viral infection

Recurrent HCV infection (93, 94) and cytomegalovirus infection (24) are risk factors for PTDM.Since hepatitis C relapse after transplantation becomes a rare phenomenon and the incidence or recurrence of nonalcoholic fatty liver disease increases, the incidence and impact patterns of PTDM could change. Current data suggest that PTDM increases the risks of infection, chronic renal failure, and biliary complications, and lowers the survival rate of recipients (25, 95). The results of Baid S et al. showed that the prevalence of PTDM in HCV(+) liver transplant recipients (64%) was significantly higher than that in HCV (–) recipients (28%), and HCV infection increased the risk of PTDM by 2.5 times (30). Bloom et al. (96) conducted a retrospective analysis of 427 kidney transplantation recipients and found that hepatitis C virus infection was an independent risk factor for PTDM. The possible reasons why HCV causes PTDM are: that HCV virus directly replicates in pancreatic islet β cells, causing dysfunction of pancreatic β cells and autoimmune destruction, resulting in insufficient insulin synthesis and secretion; HCV directly damages liver cells, resulting in insulin utilization disorders in liver cells, resulting in insulin resistance; and HCV can also affect the synthesis of insulin-related proteins by mediating the insulin signaling pathway, and ultimately affecting insulin secretion (69).

Hjelmesaeth et al. (97) reported that the incidence of PTDM in asymptomatic CMV-infected transplant recipients was significantly higher than that in CMV-uninfected transplant recipients. CMV infection increases the incidence of PTDM, which could be caused by CMV-induced or leukocyte-mediated pro-inflammatory cytokines causing pancreatic β cell damage, resulting in insufficient insulin secretion or insulin resistance, but the specific mechanism is not yet clear (69).

#### 5.3.3 Recipient, donor, and surgery-related factors

##### 5.3.3.1 Factors related to recipient

Some studies have conducted meta-analysis (5) and reported that PTDM is related to the recipient’s gender, age, HCV infection, preoperative weight, preoperative BMI, pre-transplantation FPG, family history of diabetes, HCV and other factors.

###### 5.3.3.1.1 Gender

Boktour et al. (98) reported that the recipient’s gender is associated with PTDM, and men are at higher risk than women, which may be related to gender construction, lifestyle or other social factors (99).

###### 5.3.3.1.2 Age

Abe et al. (100) reported that recipients with PTDM are older than non-PTDM patients, and the older that the recipient is, the higher that the incidence is. With age, islet β cells age and even apoptosis, insulin secretion decreases insulin resistance increases, and the risk of PTDM increases 2.2 -fold when transplant recipients are older than 45 years (44). Cosio et al. (101) analyzed the data of 2078 kidney transplantation recipients using a multivariate Cox model. It was found that patients over 45 years old at the time of transplantation were more than twice as likely as younger patients to develop PTDM. In addition, studies have also suggested that the risk of developing PTDM more than doubled for every 10-year increase in recipient age (102).

###### 5.3.3.1.3 BMI

Saliba et al. (103) have shown that recipients with a high preoperative BMI are more likely to develop PTDM than those with a low preoperative BMI. Therefore, it is recommended to appropriately control the recipient’s BMI, which is more effective in reducing the possibility of postoperative PTDM. Obesity is considered as an important independent risk factor for PTDM. Kasiske et al (63) reported that the risk of PTDM in recipients with BMI ≥ 30kg/m^2^ was significantly higher than that in recipients with BMI < 30kg/m^2^. Cosio et al. (101) conducted a retrospective analysis of 1811 kidney transplant recipients and found that elevated triglyceride levels before transplantation were closely related to the occurrence and development of PTDM. Hypertriglyceridemia is closely related to insulin resistance, so recipients with hypertriglyceridemia have an increased risk of PTDM (104).

###### 5.3.3.1.4 Pre-transplantation FPG and family history of diabetes

Pre-transplantation DM is the main factor for predicting the occurrence of PTDM (105). Driscoll et al. (106) found that pre-transplantation FPG was a risk factor for PTDM, and recipients with preoperative FPG more commonly had higher postoperative PTDM than those without FPG. Similarly, Gomes et al. (51) reported that higher FBG levels before transplantation and the occurrence of IFG before transplantation were predictive risk factors for the occurrence of PTDM, and the risk of PTDM increased with increasing FBG levels. For pre-transplantation IFG patients, 70% developed hyperglycemia one year after surgery. Li et al. (99) concluded that PTDM was associated with the recipient’s family history of diabetes, and recipients with a family history of diabetes had a significantly higher risk of PTDM than recipients without.

###### 5.3.3.1.5 HCV positive status

Gane et al. (107) suggested that HCV is a risk factor for PTDM. The incidence of PTDM in HCV-positive recipients is higher than that in HCV-negative recipients. Regular treatment of HCV-positive recipients can prevent the occurrence of PTDM. Therefore, HCV-positive recipients should be given antiviral therapy.

##### 5.3.3.2 Donor-related factors

The occurrence of PTDM is related to the donor’s gender, hepatic steatosis, and CMV positivity and to the method of transplantation (5). The incidence of PTDM in recipients with donor fatty liver degeneration was higher than that in donors without fatty liver degeneration. Nemes et al. (108) reported that PTDM is related to the donor’s gender, and men are more likely to experience PTDM than women. Ling et al. (109) suggested that donor CMV infection is a risk factor for PTDM.

##### 5.3.3.3 Surgical and postoperative factors

Fifty percent of cases with PTDM occurred within 6 months after transplantation and 75% after 12 months (110). Surgery-related risk factors include cooling time >9 hours (111), no induction therapy with basiliximab after organ transplantation, acute transplant rejection (27, 94), and other factors such as the stress response to surgery, which can impair post-transplantation liver function, thereby reducing the ability to regulate glucose homeostasis.

Acute rejection is an independent risk factor for PTDM, which is a stress response. Once acute rejection occurs, the secretion of insulin antagonistic hormones such as growth hormone, catecholamines, and glucagon in the recipient increases, and the occurrence of acute rejection requires pulse therapy with high-dose glucocorticoids, which cause the body to be in a hyperglycemic state, increasing the incidence of PTDM (112, 113).

In addition, studies have suggested that autosomal dominant polycystic kidney disease (ADPKD) is a risk factor for PTDM (114, 115). Patients with autosomal dominant polycystic kidney disease have three (3) times the risk of developing PTDM within one (1) year after kidney transplantation compared with patients without this disease (114).

## 6 How can the incidence of PTDM be reduced?

With pancreas/islet cell transplantation patients, macronutrient maldigestion is the foremost cause of progressive nutrition and metabolic deficiencies. The main ways fornutrition interventions is to prevent or treat malnutrition by ensuring adequate macronutrient and micronutrient intake and to decrease malabsorption and maldigestion (116). Maintaining nutrition balance is crucial in order to minimize the risk of cardiovascular events and metabolic complications in the kidney transplantation population (117).There is evidence that the preoperative optimization of the nutritional can improvement of short-term outcomes after liver transplantation (118)

The relevant lifestyle changes occurring after transplantation may profoundly affect glucose regulation. Before transplantation, many patients will pay special attention to their diet and deliberately control their diet, resulting in long-term insufficient calorie intake (119). Post transplantion, improved appetite and return to free food assumption lead to an increased intake of total calories and calories from carbohydrate and lipids, such as High-fat-diet(HFD),favouring weight gain and the metabolic abnormalities associated with central obesity, including diabete and dyslipidaemia (85). Concurrently, increased salt intake may favour and unmask arterial hypertension, thus further increasing cardiovascular risk in transplanted patients (119). Additionally, the use of related immunosuppressants further increases the incidence rate of postoperative diabetes. Hypercholesterolemia and hypertriglyceridemia have been reported as side effects of these drugs (120).

There are many risk factors leading to the occurrence of PTDM, among which, are modifiable risk factors that can be controlled before transplantation. Modifiable risk factors mainly include the following: obesity, glucocorticoids, CNIs, hepatitis virus infection, cytomegalovirus infection, and pre-transplantation blood glucose.

### 6.1 Obesity

Studies have suggested that abdominal circumference has good correlations with visceral obesity, insulin resistance and dyslipidemia. These conditions are closely related to the occurrence of type 2 diabetes. Therefore, it is necessary for obese patients to reduce their body weight and abdominal circumference as much as possible before surgery to reduce the occurrence of PTDM (121).

### 6.2 Glucocorticoids

Kim et al. (122) reported that compared with traditional immunotherapy (anti-rejection regimens including glucocorticoids and tacrolimus after surgery, glucocorticoids are gradually reduced and withdrawn within 2-6 months after surgery), with early steroid withdrawal protocols (combined with tacrolimus and mycophenolate mofetil anti-rejection regimen, glucocorticoids gradually reduced and withdrawn within 7 days after transplantation), the incidence of PTDM in the former is significantly increased, suggesting that early discontinuation of glucocorticoids colud reduce the incidence of PTDM. In conclusion, based on not increasing various acute and chronic immune rejection reactions, choosing an immunosuppressant regimen with individualized hormone dosages can reduce the occurrence of PTDM (69).

### 6.3 CNIs

Muduman and Liu et al. (123, 124) reported, that compared with cyclosporine A, tacrolimus can reduce adverse reactions, such as mortality, deformity rejection and hypertension after transplantation, but it leads to an increase in the incidence of PTDM. Therefore, as a commonly used immunosuppressant after transplantation, tacrolimus must be closely monitored for indicators such as plasma glucose, blood drug concentration, and renal function. In addition, there are some immunosuppressants in clinical practice, such as sirolimus, and everolimus. These drugs have a lower risk of glucose metabolism disorder than CNIs, but because of their weaker immunosuppressive effects, sirolimus and everolimus have more serious adverse effects, which is another reason why they are not as widely used as tacrolimus. However, which were generally used for maintenance therapy in the stable phase after surgery or in high-risk patients with PTDM (125).

In addition, Van Laecke et al. (28) found that the plasma Mg^2+^ levels in liver transplant recipients with diabetes were lower before surgery and within the first month after surgery, confirming that hypomagnesemia is a risk factor for PTDM. CNIs can cause a decrease in plasma Mg^2+^ levels, leading to hypomagnesemia (126). Hypomagnesemia is also a risk factor for PTDM, and its mechanism might be as follows: hypomagnesemia can reduce the activity of insulin tyrosine kinase, affect the synthesis and secretion of insulin, and cause hyperglycemia after transplantation. This phenomenon indicates that CNIs might elevate plasma glucose levels by inducing hypomagnesemia. Sinangil et al. (127) also found that the lower that the preoperative plasma Mg^2+^ concentration was, the greater that the possibility was of the occurrence of PTDM. Therefore, supplementing with magnesium before surgery and closely monitoring plasma magnesium levels after surgery can reduce the occurrence of PTDM.

### 6.4 Hepatitis virus and cytomegalovirus infection

Hum et al. (128) found that after continuous antiviral treatment in patients with diabetes and HCV infection, the plasma glucose level of the patients can be controlled to a certain extent, and the dosage of insulin can also be reduced. Therefore, patients with HCV infection can adopt continuous antiviral therapy, which may reduce the incidence of PTDM. In addition, the prevention of CMV infection in the perioperative period can reduce the occurrence of PTDM and shorten the hospitalization time of patients (69).

### 6.5 Pre-transplantation plasma glucose

Studies (51, 129) have suggested that impaired fasting plasma glucose regulation before transplantation is a harbinger of PTDM and has a certain predictability. The higher that the preoperative FPG is, the greater that the risk is of PTDM. Therefore, an active and effective control regimen should be given to patients with abnormal plasma glucose regulation before surgery (69).

## 7 Conclusion

In conclusion, PTDM is a common severe complication after organ transplantation. The risk factors for PTDM are complicated, and PTDM increases the risks of cardiovascular disease and infection. However, PTDM is often undiagnosed, underestimated, or poorly managed. Risk factors for PTDM should be evaluated during the pre-transplantation assessment, and the diagnosis of PTDM should be deferred until the recipient is on stable maintenance doses of immunosuppressive drugs and has stable transplant organ function to reduce the possibility of diabetes and avoid affecting transplant organ function, thereby improving the survival rate of transplant recipients.

## Author contributions

MG and XY contributed to conceived and designed the review. JS and ZZ did the document retrieval. JS wrote the paper. MG and XY checked the paper. All authors contributed to the article and approved the submitted version.
